# Comparative Study of Dental Custom CAD-CAM Implant Abutments and Dental Implant Stock Abutments

**DOI:** 10.3390/jcm12062128

**Published:** 2023-03-08

**Authors:** Daniel Adrian Târtea, Mihaela Ionescu, Horia Octavian Manolea, Veronica Mercuț, Eugenia Obădan, Marina Olimpia Amărăscu, Petre Costin Mărășescu, Luminița Dăguci, Sanda Mihaela Popescu

**Affiliations:** 1 Department of Oral Rehabilitation, University of Medicine and Pharmacy of Craiova, 200349 Craiova, Romaniasanda.popescu@umfcv.ro (S.M.P.); 2 Department of Medical Informatics and Biostatistics, University of Medicine and Pharmacy of Craiova, 200349 Craiova, Romania; 3 Department of Dental Materials, University of Medicine and Pharmacy of Craiova, 200349 Craiova, Romania; 4 Department of Dental Prosthetics, University of Medicine and Pharmacy of Craiova, 200349 Craiova, Romania; 5 Department of Dental Morphology, University of Medicine and Pharmacy of Craiova, 200349 Craiova, Romania; 6 Department of Dental Technology, University of Medicine and Pharmacy of Craiova, 200349 Craiova, Romania

**Keywords:** dental prostheses, CAD/CAM, dental technology, evaluation methods

## Abstract

The implementation of CAD-CAM systems in dentistry has significantly influenced the evolution of dental implantology and implant-supported prosthetics within the past three decades. Implant-supported prostheses are comfortable and aesthetic. The prosthetic abutment has also faced a rapid design evolution, from the individualization of standard stock abutments offered by various manufacturers to a modern customization process using CAD-CAM technology. This paper presents a comparative study between 20 dental custom CAD-CAM implant abutments and 20 dental implant stock abutments, based on a set of measurements performed on the digital casts obtained from 24 cases of prosthetic rehabilitation on implants. The statistical analysis (Mann–Whitney U test) revealed significant differences between these two types of abutments: the incisal margin line diameter dimensions for custom abutments were significantly improved compared to standard abutments at the cervical level (U = 343.00, z = 3.868, *p* < 0.0005) and the incisal/occlusal level (U = 352.00, z = 4.112, *p* < 0.0005), while the inclination angle of the custom abutments relative to the 0-axis was significantly smaller than that of standard abutments (U = 115.50, z = −2.286, *p* = 0.022). The use of custom abutments leads to an increase in the final size of the abutment, an improvement in the retention of the prosthetic work, and reduces the angulation of the abutment in relation to the implant axis, thus decreasing the risk of unscrewing or fracturing the dental screw.

## 1. Introduction

With the development and implementation of CAD-CAM systems in industry, followed by the significant improvement of design and production applications, the adoption of CAD-CAM in other fields of activity such as dentistry has been achieved almost naturally [1].

Dental implantology has evolved in recent years and, with the implementation of CAD-CAM systems in dentistry, implant prosthetics have also developed [2,3]. Due to the predictability of dental implants’ longevity, in more than three decades of implant-prosthetic treatment, dental implantology has offered hope to patients, obtaining stable, comfortable, and aesthetic implant-supported restorations [4]. One of the most important components of implant restauration is the prosthetic abutment.

The prosthetic abutment has seen a design evolution, starting from the individualization of stock abutments offered by manufacturers, until today, where they are customized with the CAD-CAM technology [5,6]. Before the introduction and implementation of CAD-CAM technology, there were only two types of prosthetic abutments distributed by manufacturers: stock abutments and fully calcinable or partially calcinable abutments with prefabricated interface, also known as UCLA abutments [7]. After the introduction of CAD-CAM technology, prosthetic abutments also started to be produced with the help of this technology. They can be adapted and individualized to each particular situation, offering a series of advantages, including the fact that they can be produced from different materials, especially titanium and zirconia [7].

The first customized prosthetic abutment using CAD-CAM technology was made in the United States in 2000 under the name “Atlantis Custom Abutments Technology” [8], Ossario and the company Atlantis Components registered this technology under the name “computer-aided virtual design technology for implant abutments” [9,10,11].

Before the production of custom prosthetic abutments using CAD-CAM technology by the Atlantis Company, Procera technology was developed by Nobel Biocare Implants in the mid-1990s, which initially produced all-ceramic crowns, and later applied the technology to the production of prosthetic abutments [12]. It worked on the “wax-scan-mill” principle, being a closed system, used only for Nobel Biocare implants. This technological process comprised the following phases: wax-up of the prosthetic abutment inserted into an analog by the dental technician, followed by the scanning phase, then a milling phase using ceramic blocks.

The milling of these individualized prosthetic abutments was performed using precision industrial machines at first. In the last 15 years, digital technologies used in dentistry have experienced a rapid development. Thus, the manufacturers of industrial software and milling machines oriented their interest towards the development of equipment in the field of dentistry. CAD and CAM software manufacturers have developed dental modules so that dental offices and dental laboratories can design different types of dental restorations and then manufacture them using CAM software and small milling machines in four or five axes.

Among the most popular CAD software, there are Exocad, 3Shape, or DentalWings, and among the CAM software, the most used are hyperDENT, CIMSystem-Millbox, or WorkNC. In addition to the already common materials currently milled in dental laboratories, namely zirconia, ceramic, PEEK, PMMA, or wax, manufacturers have offered the possibility of milling metal, such as Chrome-Cobalt or titanium. Last but not least, dental laboratories can currently mill customized titanium prosthetic abutments, either from prefabricated abutments also known as pre-mill abutments, or by milling directly into the titanium disc. The prefabricated abutments are sold with the interface that connects to the implant already industrially milled by the manufacturer, so that only the area of the emergence profile, the cervical line, and the prosthetic abutment are milled in the laboratory. The possibility to mill the complete abutment, including the implant interface, into the titanium disk represents an advanced feature of CAM software and it is only employed by experienced dental technicians. Currently, dental laboratories are customizing prosthetic abutments, either analogically by milling stock prosthetic abutments, or digitally by designing and milling custom abutments using CAD-CAM technology. Custom prosthetic abutments based on CAD-CAM technology have started to be used more and more, because they can be adjusted to each situation, and also because other materials besides titanium may be used, such as zirconia, ceramic, PEEK, or BioHPP [12,13].

Since the two types of abutments, stock, and customizations made by CAD-CAM technology have been used in parallel in recent years, comparative discussions have arisen regarding the advantages and disadvantages of using one type or another.

The objective of this study is to evaluate the differences between the two types of abutments, in terms of area, volume, vestibulo-oral (V-O), and mesio-distal (M-D) diameter, both in the cervical and occlusal areas. The null hypothesis is that there are no differences between stock and customized abutments in respect to their functional characteristics.

## 2. Materials and Methods

This study was carried out on the digital casts of the abutments created in the laboratory for various clinical cases of implant-prosthetic rehabilitation, from the period 2020–2022. Sample size was computed using G*Power 3.1.9.7, Heinrich Heine University Düsseldorf, Germany, considering a significance level α of 0.05, a power 1-β equal to 0.8, and a medium effect size value (since there are very few data available in the literature and with an awareness of practical significance), thus ending up with a study lot of 40 abutments. The digital casts corresponding to 20 stock prosthetic abutments that were individualized by dental technicians, and 20 customized abutments obtained through digital design and milling using CAD-CAM technology, were studied comparatively.

The stock abutments were produced by the companies Bredent Group (Senden, Germany) and Dentium Co., LTD (Seoul, Republic of Korea), and the customized CAD-CAM abutments were milled in titanium discs in laboratory using Yenadent CAM-Library on Bredent and Dentium compatible platforms [14,15]. For all patients included in this study, the implants used for oral rehabilitation required only prosthetic abutments with platforms with a diameter of 4 mm. This diameter is not dependent on the patients’ physical characteristics; therefore, the present study focuses only on technical data related to the difference between the two types of abutments, without considering other patients’ clinical data. The choice of the abutment type depended only on the criteria used to obtain the optimum time/work efficiency ratio. The stock prosthetic abutments are usually chosen by the dentist and the dental technician to provide the most suitable angulation for each individual case so that the individualization procedure at the Parallelograph does not require an excessive roughening of the volume of the prosthetic abutment.

Custom prosthetic abutments based on CAD-CAM technology are created by designing them in Exocad, and then completely milling them (the interface, emergence profile, cervical line, and the abutment) in a Titanium Magnum Hyperone disc provided by the manufacturer MESA Italia (Travagliato, Brescia, Italy), using the software CAM hyperDENT Classic (8853 Spa Pero, Italy), and the Yenadent D43 5-axis wet-milling machine (Yena, Istanbul, Turkey).

In our study, we used the Parallelograph (Pi Dental Orthoflex, Hungary), a laboratory scanner from Pi Dental (Hungary), Exocad design software v2.3 Matera (Exocad GmbH, Darmstadt, Germany), CAM hyperDENT Classic software v9.1 (FOLLOW-ME! Technology Group, Munchen, Germany), and the milling machine Yenadent D43 (Yena, Istanbul, Turkey).

The study was approved by the Ethics Committee of the University of Medicine and Pharmacy of Craiova, no. 210/10.11.2022.

### 2.1. Individualization of Stock Prosthetic Abutments

**Figure 1:**
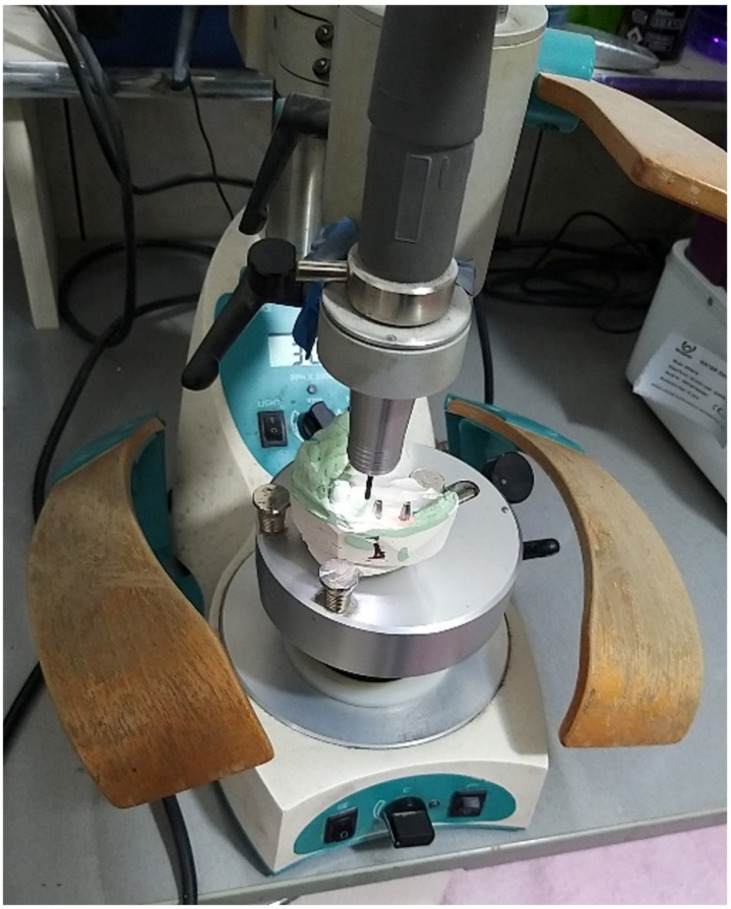
Standard abutment milling with Parallelograph Pi Dental.

The dental impressions were received in the laboratory both in an analog and digital format, and the functional working casts were either composed of plaster, or a specific 3D-printed resin. The dentist and the dental technician analyze the axis of insertion of the implant and choose a stock prosthetic abutment as close as possible to the ideal position for the insertion of the future implant-prosthetic restoration. The cast is fixed in the Parallelograph by the technician, then the grinding parameters are set, and the standard abutment is roughened to obtain the desired axis and, implicitly, the parallelism of the abutment (Figure 1).

**Figure 2:**
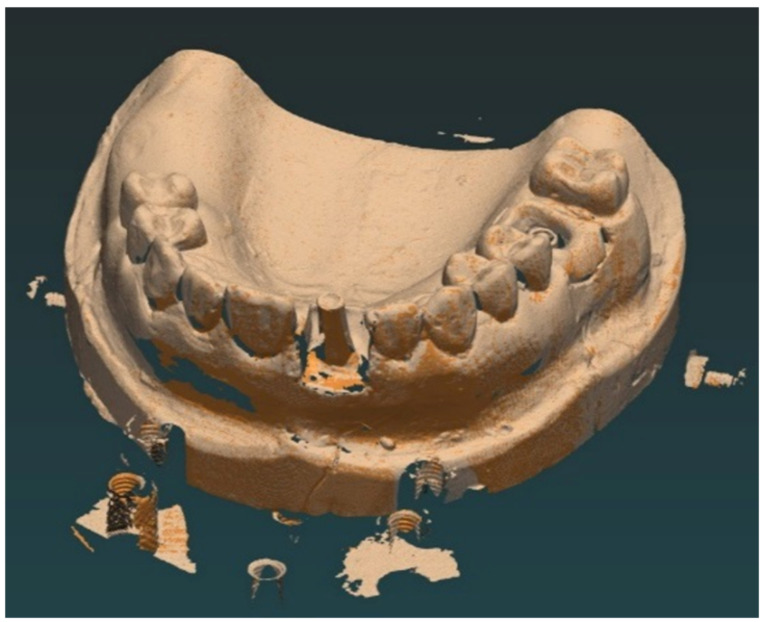
Scanned dental cast of the prepared stock abutment.

At the end, the dental technician verifies if there is enough space for the implant-prosthetic restoration, then proceeds to the stage of scanning the cast in occlusion with the fixed prosthetic abutment (Figure 2), and then designs the future superstructure in Exocad design software.

**Figure 3:**
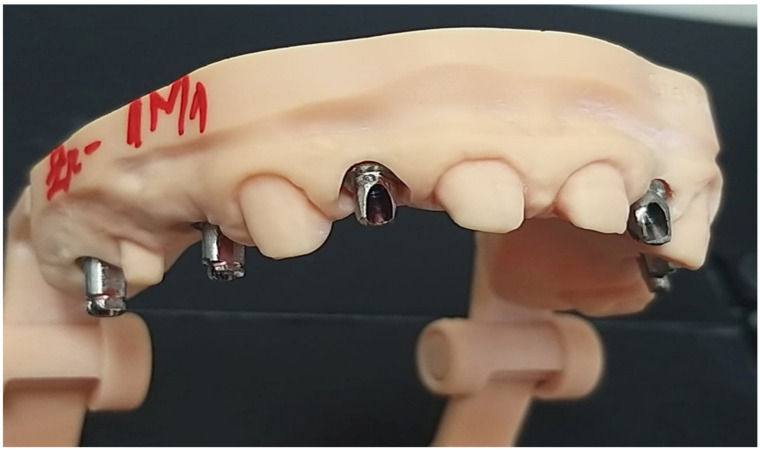
The printed working cast with the stock milled abutments, ready to be tested in the oral cavity.

These individualized standard abutments, adjusted using the Parallelograph and inserted in the implant analogs from a printed working cast, will be screwed to the implants in the patient’s oral cavity (Figure 3).

### 2.2. Individualization of Implant-Prosthetic Abutments Using CAD-CAM Technology

**Figure 4:**
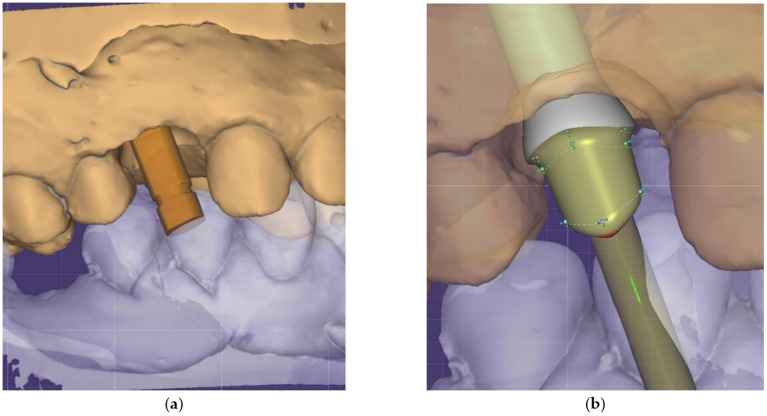

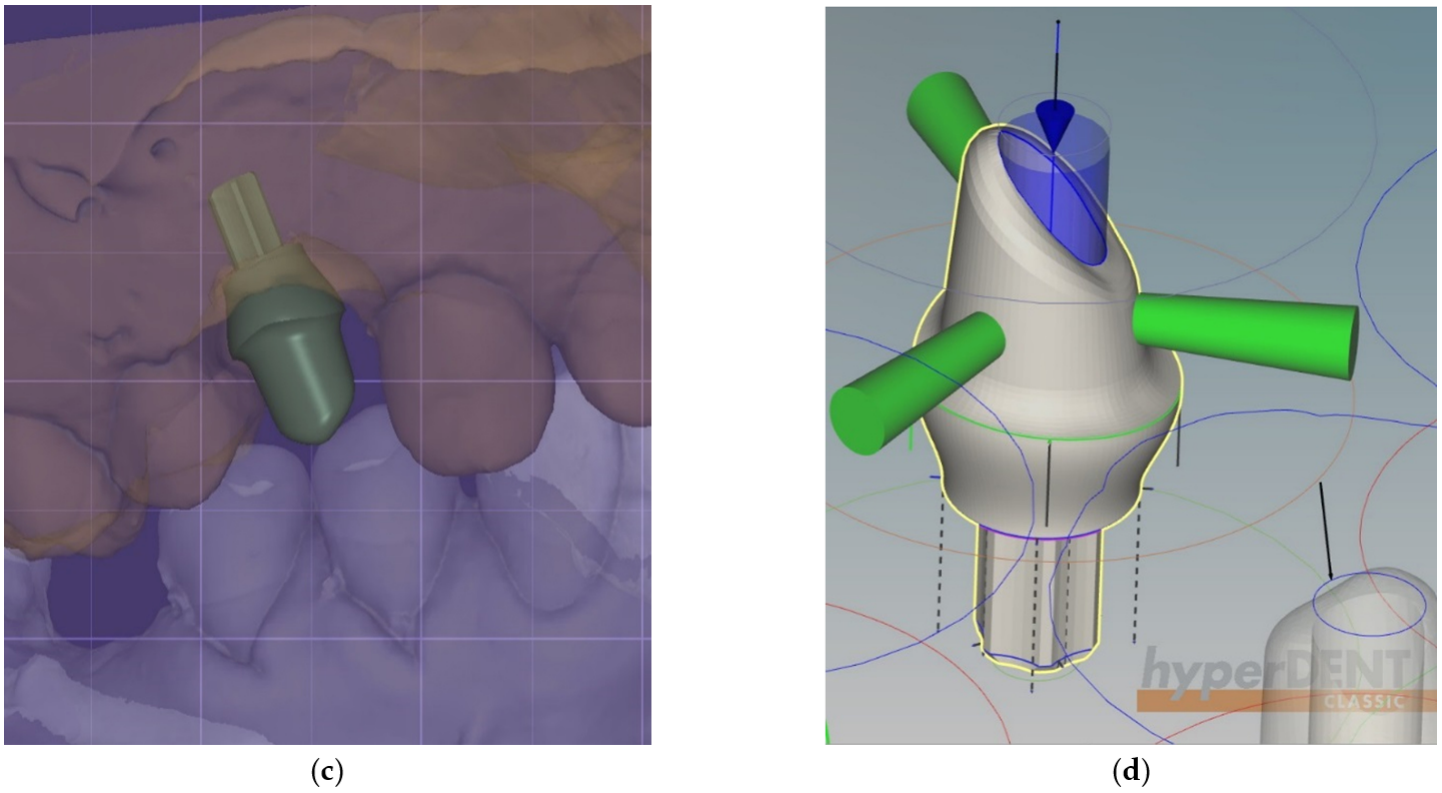
(**a**) Scanning of the *scanning abutment* to allow the virtual transfer of the implant position, taking the information from the scanning software and loading it into the Exocad design software; (**b**) the design of the prosthetic abutment; (**c**) prosthetic *abutment with connection*; (**d**) loading in the hyperDENT Classic software and computing the milling parameters.

As in the case of stock implant-prosthetic abutments, the functional working dental casts are created in the same way, only in this case, additional procedures appear, such as scanning the so-called *scanning abutment* (Figure 4a) to allow the virtual transfer of the position of the implant and to retrieve the information from the scanning software and load it into the Exocad design software. For this, a specific library that allows the design of a prosthetic *abutment with connection* (Figure 4b) was used, which was then loaded into the CAM software (Figure 4c) that allows direct milling of the connection in a titanium disc (Titanium Magnum Hyperone, Mesa di Salla Giacomo, Averolda e Finiletti, Italy) (Figure 4d).

The abutments obtained by this milling procedure are removed from the disc, and the connectors are removed by fine machining. Then, the abutments are scanned with the individual casts in occlusion. Afterwards, the design of future prosthetic restorations is created with the design software. Between these two types of individualized implant-prosthetic abutments, real differences in appearance and volume may be observed. The current use of the two types of implant-prosthetic abutments makes a comparative analysis useful in terms of area, volume, and V-O and M-D diameters, both in the cervical and occlusal areas.

### 2.3. Measurements

For each digital cast of the abutment, the following parameters were measured: surface area (expressed in mm^2^), volume (expressed in mm^3^), height (expressed in mm), width (expressed in mm), length (expressed in mm), and angle (expressed in degrees).

The *Measurement Tool* provided by the Exocad software was used to obtain these measurements. The area, volume, height, and V-O and M-D diameters in the cervical area were automatically computed by the software, while the diameters in the occlusal area, V-O and M-D, and the inclination to the 0-axis of the implant were performed manually, which may hold a subjective value.

### 2.4. Statistical Analysis

Measurements were initially processed using Microsoft Excel (San Francisco, CA, USA), allowing the distribution of the study group in subgroups (standard group and custom group). Continuous variables were defined as mean ± standard deviation and were compared using Mann–Whitney U test for non-Gaussian distributions (identified using the Shapiro–Wilk test). All statistical tests were completed using Statistical Package for Social Sciences (SPSS), version 20 (IBM Corp., Armonk, NY, USA). The value of α threshold was set to 5%, and the value *p* < 0.05 was considered statistically significant.

## 3. Results

This retrospective study compared digital casts made for 20 stock milled prosthetic abutments and 20 digital casts of the custom abutments obtained by digital design and milling using CAD-CAM technology.

**Figure 5:**
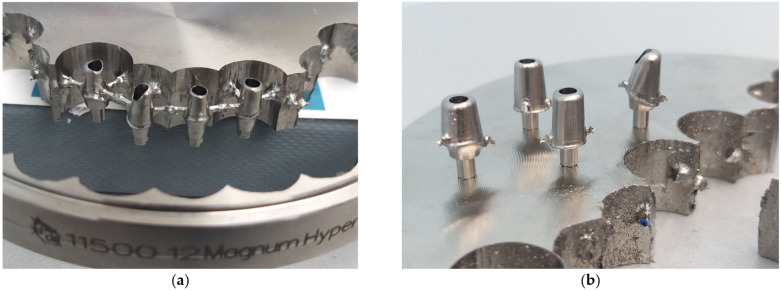
Example of customized prosthetic abutments milled into disc by Titanium Magnum Hyperone: (**a**) aspect after milling; (**b**) aspect after removal from the disc.

The aspect of the custom abutments after milling is presented in Figure 5.

**Figure 6:**
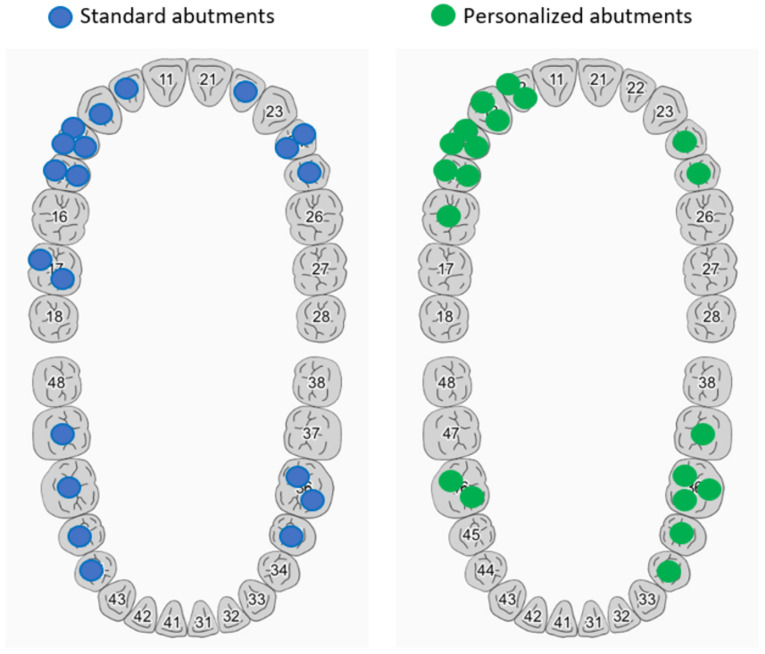
Abutments’ distribution on mandible/maxillary.

Distribution of teeth restored with implant restorations is emphasized in Table 1 and Figure 6.

**Figure 7:**
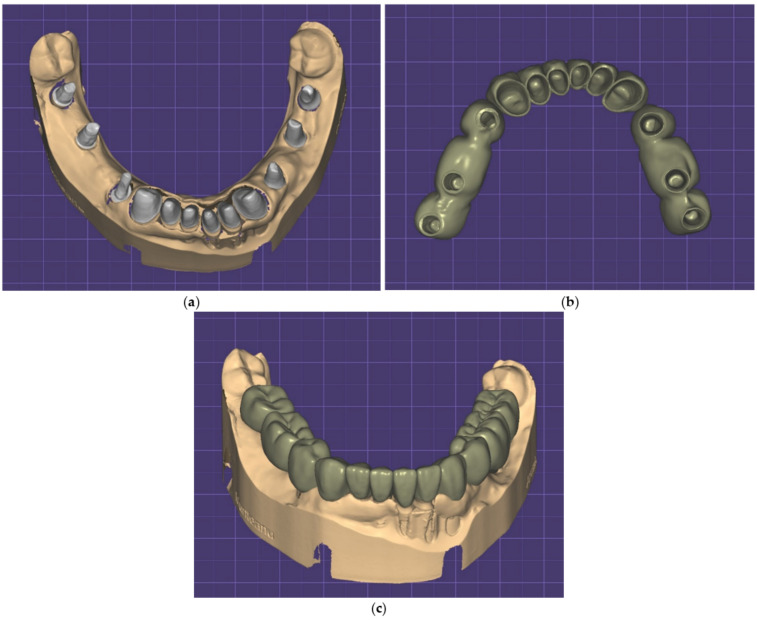
(**a**) Aspect of the scanned working cast, with stock milled abutments and customized CAD-CAM abutments; Bredent standard abutments 44, 46, 47; custom CAD-CAM abutments with Bredent connection 34, 36, 37; (**b**,**c**) the design of the front dental prosthetic restoration and the two lateral implant-prosthetic restorations.

An example of a case in which the same patient received both stock milled abutments and customized CAD-CAM implant-prosthetic abutments is presented in Figure 7. For the implants positioned in place of teeth 44, 46, and 47, Bredent stock milled abutments were used, and for the implants positioned in place of teeth 34, 36, and 37, customized CAD-CAM abutments with Bredent connection were used. The image shows a higher emergence profile for CAD-CAM abutments.

The parameters compared for the two sets of abutments were surface area (mm^2^), surface volume (mm^3^), height, ∅ at the V-O bundle, ∅ at the V-O incisal edge, ∅ at the M-D bundle, ∅ at the M-D incisal edge, and front abutment inclination of axis 0.

Group distributions were analyzed using a Mann–Whitney U test to determine whether there were differences in measured parameters between stock and custom abutments (Table 2). Distributions of these parameters for standard abutments and custom abutments were not similar, as assessed by visual inspection.

For stock abutments, surface area had values between 64.19 mm and 142.5 mm, with an average value of 102.67 ± 26, while the custom abutments’ minimum value was 52.61 and maximum value was 125.2, with an average value of 78.95 ± 18.09. Surface areas for stock abutments were statistically significantly higher than for custom abutments, U = 92.00, z = −2.921, *p* = 0.003. Similar results were obtained for height measurements, as custom abutments had statistically significantly lower heights than stock abutments, U = 85.00, z = −3.111, *p* = 0.002. However, volume measurements revealed that stock abutments presented lower values than custom abutments, still overall values were not statistically significantly different, U = 250, z = 1.353, *p* = 0.176.

Custom abutments presented higher values for cervical diameters compared to stock abutments, both for V-O and M-D measurements, but without any statistically significant differences (U = 221, z = 0.568, *p* = 0.570 for V-O, and U = 271.00, z = 1.921, *p* = 0.055 for M-D). On the other hand, analysis of margin line diameters revealed statistically significantly higher values for custom abutments compared to stock abutments, U = 343.00, z = 3.868, *p* < 0.0005 for V-O and U = 352.00, z = 4.112, *p* < 0.0005 for M-D.

Inclination angles for custom abutments were statistically significantly lower than for stock abutments, U = 115.50, z = −2.286, *p* = 0.022.

## 4. Discussion

The results of the present study show that titanium implant abutments customized by the CAD-CAM method present several advantages compared to stock titanium ones customized by milling. These advantages are that mesio-distal dimensions of custom abutments (both at the margin line and incisal/occlusal level) are significantly greater than those of stock abutments and the inclination of CAD-CAM custom abutments to the 0-axis is significantly less than in the case of stock abutments. These advantages translate into a much smaller conicity of the customized CAD-CAM abutments than the standard ones individualized by milling, in the conditions where a good parallelism of the implant abutments is obtained within an implant-prosthetic restoration made with support on several implants. The angulation of custom CAD-CAM abutments will be less than that of stock angled abutments, as demonstrated by the inclination to the 0-axis of the abutments, which is significantly smaller in the case of custom CAD-CAM abutments. The use of customized CAD-CAM implant abutments offers several possibilities for implant-prosthetic rehabilitation: both by cementation and by screwing.

When discussing two different types of implant-prosthetic abutments from the same material, there is a tendency to show the advantages of using one over the other.

In a study regarding the cementation of ceramic crowns on custom implant abutments, Edelhoff et al. claim that individualized implant-prosthetic abutments with the help of CAD-CAM technology represent an advantage in terms of designing the geometry of the abutment, with the drawing of the margin line exactly where it is desired, which significantly reduces the possibility of remaining cement residues in the peri-implant sulcus [13].

Regarding the peri-implant sulcus, a study was also written by Lops et al. in which it is shown that the soft tissue retraction was greater in the case of individualized standard abutments, and in the case of CAD-CAM implant-prosthetic abutments, the authors even found an increase in the soft tissue [16].

Another study conducted by I.M. Valsan et al. regarding the individualization of ceramic implant abutments through CAD-CAM technology shows that they present a real benefit because they can achieve an excellent adaptation to the package, avoiding sharp edges. The authors claim that custom CAD-CAM abutments offer a biological advantage because they perform the function of being in contact with soft tissue, whereas stock individualized abutments do not, with the dental crown fulfilling this function [17].

Another important aspect is the unscrewing or fracturing of the screw. Paek et al. showed that between the two types of prosthetic abutments there are no significant differences regarding the occurrence of screw fractures [7]. The forces applied by the authors were between 10 N and 250 N, much less than human masticatory forces (150–354 N according to Takaki et al., 2014) [18]. There are situations where masticatory forces on implant-prosthetic restorations are much higher, such as in the example given by Al-Omiri et al., 2014, in which case forces of 577.9 N were recorded on part of the implant-prosthetic restoration, and 595.1 N on the contra-lateral dentate area [19]. One of the reasons for screw fracture is the way in which the implant-prosthetic abutment connects to the implant, without micro-movements. In a study by Apicella et al., to evaluate the adaptation of the connection to the implant, in which they used radiography and electron microscopy (SEM—scanning electron microscopy), the result was that there were no significant differences between the two types of implant-prosthetic abutments [20]. However, these differences are evidently in favor of stock abutments, only if CAD-CAM implant-prosthetic abutments are produced by laser sintering using a 3D printer [21].

In the case of both types of implant-prosthetic abutments (standard and customized CAD-CAM), one of the biggest causes of failure is fracture and loss of the screw. The two most likely causes of abutment screw loosening are excessive bending of the joint and the settling effect of the screw threading surfaces [22]. Bolt loosening occurs when the joint separation forces acting on the bolt joint are greater than the tightening forces holding the bolt unit together. Excessive forces can cause slippage between the screw threads and the bore, resulting in the loss of preload and loosening of the screw [23]. The mechanism of the settling effect is represented by the fact that any milled surface has several degrees of micro-roughness. When there is a dynamic load, micro-movements can bring two surfaces of the screw closer together. This means that screw loosening can occur when the total settling effect is greater than the elastic elongation effect of the screw [24].

There are authors who believe that standard abutments represent an optimal solution because they are not expensive, save time, and avoid the phenomenon of corrosion that can be found in some alloys from which implant-prosthetic abutments are milled [25]. Another advantage of standard abutments comes from the fact that the industrial production process is always standardized in terms of product quality to ensure biocompatibility of the materials used [26].

However, custom CAD-CAM implant abutments present several advantages compared to stock individualized abutments, especially if we refer to the design of the emergence profile. There are clinical situations of implant-prosthetic rehabilitation in which a generous emergence profile cannot be designed in the design software if a healing abutment specific to the edentulous space was not used in the treatment plan. This is also the case of our study because we only rarely benefited from the opportunity to design an emergence profile that would allow the dental laboratory to produce an implant-prosthetic abutment with a semi-physiognomic support and appearance. Clinical studies show that by using gingiva formers suitable for the edentulous space, whether they are serially manufactured, or CAD-CAM-made, the prognosis of successful aesthetic and functional implant-prosthetic restorations is very high [27]. Additionally, the use of these gingival healing abutments leaves the possibility of using CAD-CAM implant-prosthetic abutments in the following stages that will be able to improve the support of the interdental papilla, eliminating its retraction effect [28].

The limits of this study are related to the small number of abutments and the lack of associated patients’ data. It should be emphasized that the present research provided measurements of abutments following the design phase; future research includes the clinical phase and patients’ follow-up in order to determine the condition of the abutments’ surrounding tissue and long-term resistance to fractures.

## 5. Conclusions

Custom implant abutments have significantly better values than stock abutments for parameters such as incisal margin diameter and abutment inclination to 0-axis. These parameters improve the final shape of the prostheses, the stability of the implant-supported prostheses, and transmission of occlusal forces to the implant.

## Figures and Tables

**Table 1:** Restored teeth distribution according to location.

Tooth Abutment	12	13	14	15	16	17	22	24	25	34	35	36	37	44	45	46	47
Standard	1	1	3	2	1	2	1	2	1	1	1	2	1	1	1	1	1
Custom	2	2	3	2	0	0	0	1	1	0	1	3	0	0	0	2	0
Total	3	3	6	4	1	2	1	3	2	1	2	5	1	1	1	3	1

**Table 2:** Statistical comparisons between stock abutments and custom abutments.

Parameter	Standard (Stock)		Custom		*p* *
	Mean ± SD	Mean Rank	Mean ± SD	Mean Rank	
Surface area	102.67 ± 25.96	98.20	78.95 ± 18.09	77.08	**0.003**
Volume	60.43 ± 24.70	54.99	70.93 ± 24.92	63.31	0.176
Height	8.05 ± 1.57	7.93	6.49 ± 1.00	6.34	**0.002**
Margin line ∅ to V-O	4.94 ± 0.65	4.97	5.12 ± 0.77	4.84	0.570
Incisal margin ∅ to V-O	2.09 ± 1.17	1.53	3.71 ± 0.96	3.91	**<0.0005**
Margin line ∅ to M-D	4.82 ± 0.39	4.76	5.28 ± 0.79	5.24	0.055
Incisal margin ∅ to M-D	2.61 ± 0.43	2.70	3.60 ± 0.74	3.49	**<0.0005**
The inclination of the abutment relative to the 0 axis	16.50 ± 11.31	14.55	9.95 ± 11.63	4.65	**0.022**

* Mann–Whitney U test. Bold values represent statistically significant results.

## Data Availability

The authors declare that the data of this research are available from the corresponding authors upon reasonable request.
